# Bee Venom Protects against Rotenone-Induced Cell Death in NSC34 Motor Neuron Cells

**DOI:** 10.3390/toxins7093715

**Published:** 2015-09-21

**Authors:** So Young Jung, Kang-Woo Lee, Sun-Mi Choi, Eun Jin Yang

**Affiliations:** 1 Department of Medical Research, Korea Institute of Oriental Medicine, 483 Expo-ro, Yuseong-gu, Daejeon 305-811, Korea; E-Mails: syzzim84@gmail.com (S.Y.J.); neurokangwoo@kaist.ac.kr (K.-W.L.); 2 Executive Director of R&D, Korea Institute of Oriental Medicine, 483 Expo-ro, Yuseong-gu, Daejeon 305-811, Korea; E-Mail: smchoi@kiom.re.kr; 3 Department of Clinical Research, Korea Institute of Oriental Medicine, 483 Expo-ro, Yuseong-gu, Daejeon 305-811, Korea

**Keywords:** bee venom (BV), NSC34 motor neuron cell, rotenone, phospho-JNK, cleaved caspase-3

## Abstract

Rotenone, an inhibitor of mitochondrial complex I of the mitochondrial respiratory chain, is known to elevate mitochondrial reactive oxygen species and induce apoptosis via activation of the caspase-3 pathway. Bee venom (BV) extracted from honey bees has been widely used in oriental medicine and contains melittin, apamin, adolapin, mast cell-degranulating peptide, and phospholipase A_2_. In this study, we tested the effects of BV on neuronal cell death by examining rotenone-induced mitochondrial dysfunction. NSC34 motor neuron cells were pretreated with 2.5 μg/mL BV and stimulated with 10 μM rotenone to induce cell toxicity. We assessed cell death by Western blotting using specific antibodies, such as phospho-ERK1/2, phospho-JNK, and cleaved capase-3 and performed an MTT assay for evaluation of cell death and mitochondria staining. Pretreatment with 2.5 μg/mL BV had a neuroprotective effect against 10 μM rotenone-induced cell death in NSC34 motor neuron cells. Pre-treatment with BV significantly enhanced cell viability and ameliorated mitochondrial impairment in rotenone-treated cellular model. Moreover, BV treatment inhibited the activation of JNK signaling and cleaved caspase-3 related to cell death and increased ERK phosphorylation involved in cell survival in rotenone-treated NSC34 motor neuron cells. Taken together, we suggest that BV treatment can be useful for protection of neurons against oxidative stress or neurotoxin-induced cell death.

## 1. Introduction

Rotenone is a naturally-occurring plant compound and a specific inhibitor of complex I of the mitochondrial respiration chain. It had been found to induce cell death in a variety of cells [1]. Features include mitochondrial impairment, microglial activation, oxidative damage, dopaminergic degeneration, and l-dopa-responsive motor deficit. Rotenone was thought to elevate mitochondrial reactive oxygen species production [1], decreasing cellular ATP levels [2] and mitochondrial membrane potential [3]. Other reports have indicated that rotenone induces apoptosis via an increase in mitochondria reactive oxygen species production and neurotoxicity associated with increased levels of caspase family gene expression [4] in PC12 cells.

Bee venom (BV) is known to be a very complex mixture of active peptides including melittin, phospholipase A_2_, apamin, adolapin, and mast cell-degranulating peptide [5]. Apamin, phospholipase A_2_ and melittin had a neuroprotective effect [6,7,8] and adolapin had an anti-inflammatory effect [9].

Many experimental studies on the biological activities of BV have been reported and its anti-inflammatory effects, as well as its activities in relieving pain of rheumatoid arthritis and in immune modulation, have been described [10,11,12,13]. In addition, BV has also been reported to enhance activation of the apoptotic signaling pathway in experimental osteosarcoma, breast cancer, and lung cancer cell lines [14,15,16]. Although previous studies have clearly demonstrated that BV possesses anti-proliferative and pro-apoptotic effects, those studies have primarily been performed in cancer cell lines. Recently, Doo *et al.* have shown that BV protected neuronal cells against MPP^+^-induced apoptotic cell death via activation of PI3K/Akt-mediated signaling and inhibition of cell death signaling [17].

Therefore, in this study, we investigated the effects of BV on rotenone-induced cell toxicity in NSC34 motor neuron cells. The MAPK family is known to regulate neuronal survival and death [18,19,20]; ERK1/2 is activated by growth factors, whereas JNKs are activated by cell stress-induced signaling. We examined the effect of rotenone on the activation of JNK and ERK1/2 related to cell death and cell survival, respectively. In our previous study, we demonstrated that BV had a neuro-protective effect against glutamate-induced toxicity via inhibition of the expression of phospho-JNK and phopho-ERK in neuronal cells [21]. We report that pretreatment of BV significantly attenuated rotenone-mediated toxicity via inhibition of the activation of c-Jun *N*-terminal kinase (JNK) and an increase of extracellular signal-regulated kinase (ERK) signaling pathway. In addition, pretreatment with BV significantly inhibited mitochondria impairment and the expression of cleaved caspase-3 in NSC34 neuronal cells. The present results may have clinical implications and suggest that BV may be a potential treatment for the prevention of oxidative stress-induced cell death in neurodegenerative diseases.

## 2. Materials and Methods

### 2.1. Cell Culture

The motor neuron cell line NSC34 was obtained from Cellutions Biosystems Inc. (Toronto, ON, Canada). Cells were cultured in Dulbecco’s modified Eagle’s medium (DMEM) supplemented with 10% FBS, 100 U/mL penicillin, and 100 µg/mL streptomycin at 37 °C with 5% CO_2_. Cells were subcultured in a fresh culture dish when growth reached 70%–90% confluence (*i.e.*, every 2–3 days) as recommended by Cellutions Biosystems Inc. In all experiments, cells were incubated in the presence or absence of 2.5 µg/mL of BV before the addition of 10 μM rotenone to the culture media.

### 2.2. Cell Viability Assay

Cell viability was determined by a modified MTT (3-[4,5-dimethyl-thiazol-2-yl]-2,5-diphenyltetrazolium bromide) reduction assay*.* This assay is based on the ability of active mitochondrial dehydrogenase to convert dissolved MTT into water-insoluble purple formazan crystals. NSC34 motor neuron cells were plated in 96-well plates (2 *×* 10^4^ cells/well). After 24 h, the cells were treated with the indicated concentration of BV for 24 h prior to 10 μM rotenone treatment for 24 h. Briefly, MTT was added to each well at a final concentration of 0.5 mg/mL, and the plates were incubated for 1 h at 37 °C. After removing the culture medium, DMSO was added, and the plates were shaken for 10 min to solubilize the formazan reaction product. The absorbance at 570 nm was measured using a microplate reader (Bio-rad, Hercules, CA, USA).

### 2.3. Preparation of Primary Cortical Neuronal Culture

Mixed primary cortical neuronal cells were prepared from embryonic day 15 (E15) ICR mouse embryos. Briefly, the cortical region of mouse brain was dissected and cleaned of meningeal tissue, minced, and dissociated mechanically by flamed polished Pasteur pipettes in minimal essential medium (MEM). Dissociated cortical cells were then plated in Neurobasal medium with B-27 supplement, 5% FBS (Gibco, Grand Island, NY, USA), 5% horse serum, and 2 mM glutamine onto laminin- and poly-d-lysine-coated 12-well plates. Primary cortical cultures at 14 days *in vitro* (DIV) were used.

### 2.4. Western Blot

Cells were washed twice with ice-cold phosphate-buffered saline and harvested into 1.5 mL tube. Cells were lysed with lysis buffer containing 50 mM Tris HCl, pH 7.4, 1% NP-40, 0.1% SDS, 150 mM NaCl, and the Complete Mini Protease Inhibitor Cocktail (Roche, Basel, Switzerland). The protein concentration was measured with a BCA Protein Assay Kit (Pierce, Rockford, IL, USA). Extracted samples (20 μg total protein per lane) were separated using SDS-polyacrylamide gel electrophoresis (SDS-PAGE) and then transferred to nitrocellulose membranes (Whatman, Lawrence, KS, USA). The membranes were blocked with 5% skim milk to prevent nonspecific protein binding and incubated with primary antibodies against p-ERK (1:1000, cell signaling), p-JNK (1:1000, cell signaling), total ERK (1:1000, cell signaling), total JNK (1:1000, cell signaling), α-tubulin (1:5000, Abcam, Cambridge, MA, USA), and cleaved caspase-3 (1:1000, cell signaling) in 5% skim milk overnight. After washing three times with TBS-T (pH7.5, 1 M Tris-HCl, 1.5 M NaCl, 0.5% tween-20), the membranes were hybridized with horseradish peroxidase-conjugated secondary antibodies for 1 h. Following five washes with TBS-T, specific protein bands were detected using the SuperSignal West Femto Chemiluminescent Substrate (Pierce, IL, USA) and enhanced chemiluminescence reagents (Amersham Pharmacia, Piscataway, NJ, USA). α-tubulin was used as an internal control to normalize protein loading. Protein bands were detected and analyzed using the FusionSL4-imaging system. Quantification of the blotting bands was performed using Bioprofil (Bio-1D version 15.01, Eberhardzell, Germany).

### 2.5. Mitochondria Staining

MitoTracker Red CMXRos (Molecular Probes, Eugene, OR, USA) is a red fluorescent dye that stains mitochondria in live cells. NSC34 motor neuron cells were plated in 12-well plates (5 *×* 10^4^ cells/well). After 24 h of cell seeding, the cells were treated with the indicated concentration of BV for 24 h prior to 10 μM rotenone treatment for 24 h. Briefly, cells were stained with MitoTracker at a final concentration of 100 nM according to the standard protocol. The cells were fixed with 4% paraformaldehyde at room temperature, and then washed with phosphate-buffered saline twice. The cover slip was fixed using Fluoromount to the slide glass.

### 2.6. Data Analysis

Data are expressed as means ±S.E.M. Comparisons were evaluated by one-way analysis of variance (ANOVA) and followed by Newman-keuls post-hoc test for multiple comparisons using GraphPad Prism 5.0 (GraphPad software, La Jolla, CA, USA). Probability values less than 0.05 were considered statistically significant.

## 3. Results

### 3.1. Effect of Bee Venom on Rotenone-Induced Cell Death in NSC34 Cells

**Figure 1:**
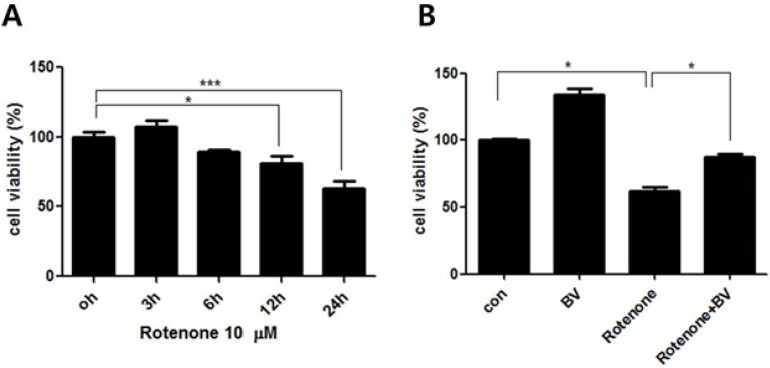
BV pretreatment prevents rotenone-induced cytotoxicity in NSC34 neuronal cells. (**A**) NSC34 cells were treated with 10 µM rotenone for 0, 3, 6, 12, and 24 h and cell viability was determined using MTT assay. 10 µM rotenone treatment induced time-dependent cytotoxicity in NSC34 cells; (**B**) NSC34 cells were treated with 2.5 µg/mL BV prior to stimulation with 10 µM rotenone for 24 h. BV pretreatment prevented 10 µM rotenone-induced cytotoxicity in NSC34 cells. The values shown are the means ±S.E.M. of data obtained from three independent experiments. * *p* < 0.05, *** *p* < 0.001.

To examine the cytotoxicity of NSC34 cells after rotenone treatment, we incubated NSC34 motor neuron cells with 10 μM rotenone for various time periods (0, 3, 6, 12, and 24 h). Our results showed that rotenone decreased cell survival in a time-dependent manner (Figure 1A). Compared to untreated NSC34 cells, 10 μM rotenone treatment for 24 h resulted in a 37% decrease in cell viability. To investigate whether BV attenuates rotenone-induced cytotoxicity, we performed a cell viability test on the cells treated with 10 μM rotenone for 24 h with or without 2.5 µg/mL BV pretreatment. BV pretreatment prevented cell death induced by 10 μM rotenone, with about 29% protection for a dose of 2.5 µg/mL BV compared to rotenone-treated cells (Figure 1B). These results indicate that BV pretreatment had a protective effect against rotenone-induced cytotoxicity.

### 3.2. Bee Venom Affects ERK and JNK Phosphorylation

**Figure 2:**
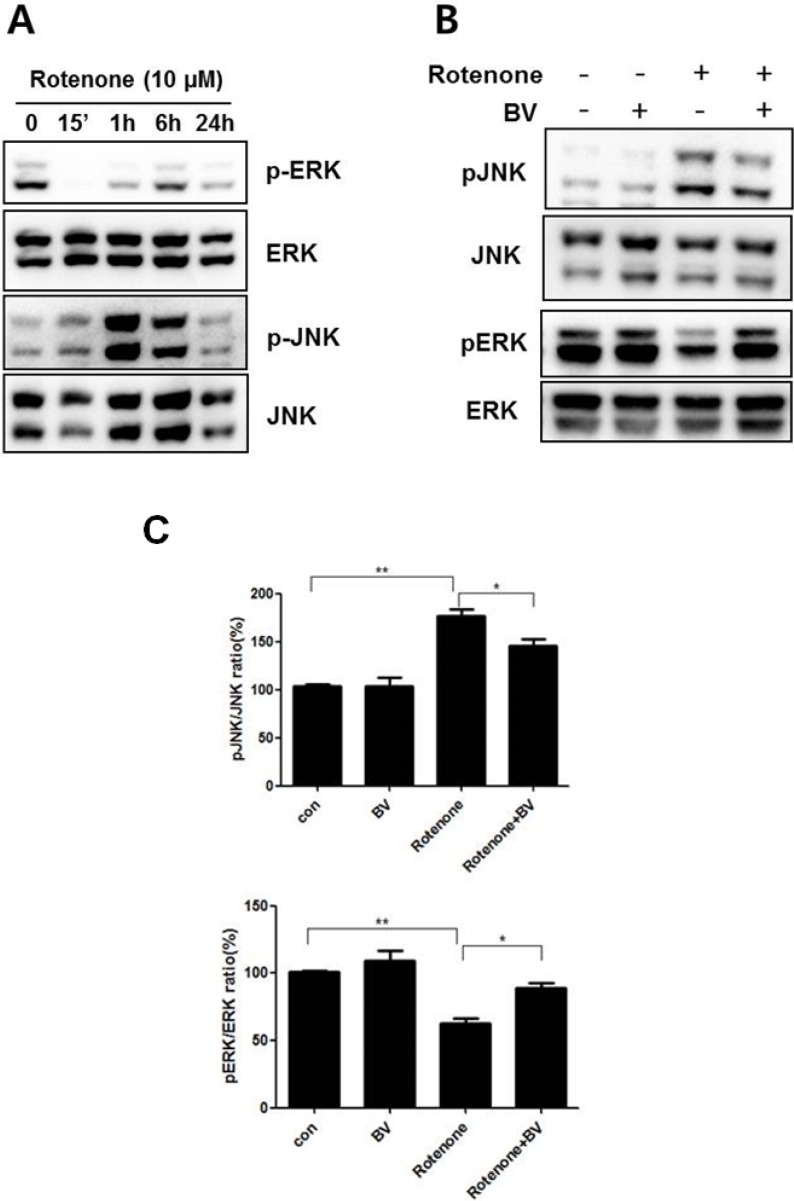
BV pretreatment regulates the activation of rotenone-mediated signaling in NSC34 neuronal cells. Effect of rotenone on the phosphorylation of the MAPK proteins ERK and JNK in NSC34 cells. (**A**) NSC34 cells were treated with 10 µM rotenone for the indicated time. Total cell lysates were separated with SDS-PAGE and Western blots were performed using anti-phospho JNK, anti-phospho ERK1/2, JNK, and ERK antibodies; (**B**) NSC34 cells were pretreated with 2.5 μg/mL BV for 24 h and then stimulated with 10 μM rotenone for 1 h in the presence or absence of BV. Western blots were performed with specific antibodies, including those for the phosphorylated forms of ERK and JNK. Total ERK and JNK were used as loading controls for the cell lysates; (**C**) Immune blots were quantified with the relative phospho-/nonphospho ratio. The values shown are the means ±S.E.M. of data obtained from three independent experiments. * *p* < 0.05, ** *p* < 0.01.

For this study, NSC34 cells were treated with 10 μM rotenone for various time periods (0, 0.25, 6, and 24 h). JNK phosphorylation, indicating JNK activation, increased after 1 h of rotenone treatment in NSC34 cells. However, the expression of ERK1/2 phosphorylation, which promoted cell survival, was significantly reduced at 15 min by rotenone treatment compared to untreated cells (Figure 2A). Both significant increase in JNK phosphorylation and decrease in ERK phosphorylation were observed after 1 h of rotenone treatment in NSC34 cells. To examine the BV effect on cell death signaling, we pretreated with 2.5 μg/mL BV for 24 h and then stimulated with 10 μM rotenone for 1 h in the presence or absence of BV. As shown in Figure 2B,C, pretreatment with BV attenuated by 1.2 fold JNK phosphorylation compared to rotenone-treated cells. In addition, BV pretreatment recovered by 1.4 fold ERK under-phosphorylation compared rotenone-treated NSC34 cells (Figure 2B,C). These results suggest that BV pretreatment could prevent oxidative stress induced-neuronal cell death by inhibiting cell death-related MAPK signaling.

### 3.3. Bee Venom Attenuates Rotenone- Induced Caspase-3 Activation in NSC34 Neuronal Cells

**Figure 3:**
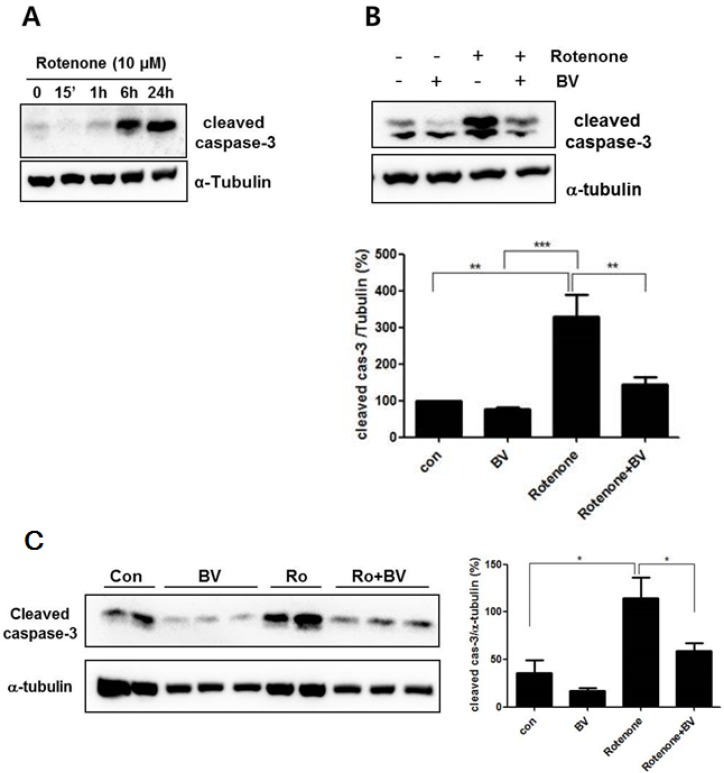
BV inhibits the expression of cleaved caspase-3 in NSC34 cells. (**A**) Expression of cleaved caspase-3 proteins was detected in rotenone-induced NSC34 cells. NSC34 cells incubated with 10 µM rotenone for the indicated time periods. The loading control for the cell lysates was determined by re-probing the membranes with α-tubulin antibody; (**B**) BV attenuates the expression level of cleaved caspase-3 protein in NSC34 neuronal cells. NSC34 cells were pretreated with 2.5 µg/mL BV for 24 h and then stimulated with 10 μM rotenone for 24 h in the presence or absence of BV; (**C**) BV inhibits the expression of cleaved caspase-3 protein in rotenone-treated in primary cortical neuronal cells system. BV pretreated with 2.5 µg/mL for 24 h and stimulated with 10 μM rotenone for 24 h in the presence or absence of BV in primary cortical neuronal cells. The loading control for the cell lysates was determined by re-probing the membranes with α-tubulin antibody. The values shown are the means ±S.E.M. of data obtained from three independent experiments. * *p* < 0.05, ** *p* < 0.01, *** compared to * and ** and indicates *p* < 0.001.

To investigate the effects of BV on oxidative stress-induced mitochondria dysfunction, capase-3 activation was assessed after exposure to rotenone in NSC34 neuronal cells. The expression level of cleaved caspase-3 was significantly increased after 6 h of 10 μM rotenone treatment compared to untreated NSC34 neuronal cells (Figure 3A). However, pretreatment with 2.5 μg/mL BV suppressed by two-fold the increase of caspase-3 activation induced by rotenone treatment compared with rotenone-treated NSC34 cells (Figure 3B,C). To confirm BV effects against rotenone, we performed the same experiment with primary cortical neuronal cells. Consistent with the result in NSC34 cell line, 10 μM rotenone treatment induced the activation of caspase-3 but 2.5 μg/mL BV treatment reduced rotenone-induced cleaved caspase-3 expression. Those results suggest that BV pretreatment could prevent oxidative stress-induced mitochondria dysfunction.

### 3.4. Bee Venom Suppresses Rotenone-Mediated Mitochondrial Impairment

**Figure 4:**
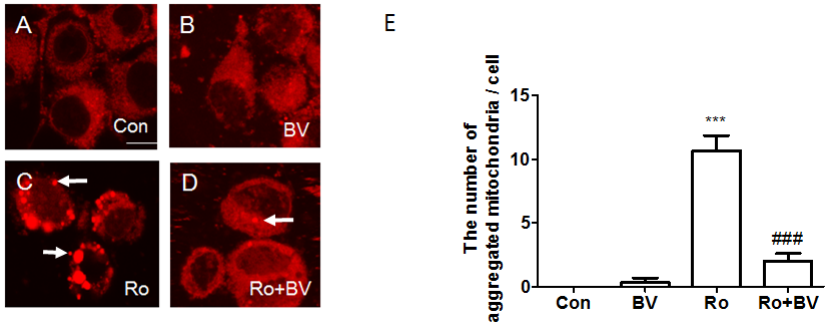
Confocal microscopy images of mitochondria using Mitotracker^®^ Red. Mitochondria stained with Mitotracker^®^ Red in NSC 34 cells untreated (**A**), treated with 2.5 µg/mL BV for 24 h (**B**) or 10 µM rotenone for 24 h (**C**), pretreated with 2.5 µg/mL BV for 24 h and 10 µM rotenone for 24 h (**D**). Arrows indicate aggregated mitochondria. BV pretreatment reduces mictochondria impairment induced by rotenone treatment in NSC34 cells. The bar indicates 200 μm. (**E**) Quantification of the number of aggregated mitochondria in a cell of 10 microscopic visual fields randomly selected. The values shown are the means ±S.E.M. of data obtained from three independent experiments. *** and ### indicate *p* < 0.001. *** compared to Con and ### compared to Ro. Con: control, BV: BV-treated cell, Ro: rotenone-treated cell, and Ro + BV: BV pretreated-cells prior to rotenone treatment.

To evaluate the effect of BV pretreatment on rotenone-induced mitochondria alteration, we stained neuronal cells with Mitotracker^®^ Red probes that passively diffuse across the plasma membrane and accumulate mitochondria of live cells and observed mitochondria using confocal microscopy. As shown in Figure 4, mitochondria showed broad cytoplasmic distribution in both control and BV-treated NSC34 neuronal cells. However, 10 μM rotenone treatment for 24 h induced aggregated mitochondria in neuronal cells (Figure 4C). Interestingly, pretreatment with 2.5 μg/mL BV inhibited mitochondrial aggregation resulting from rotenone treatment (Figure 4D,E). These findings suggest that BV pretreatment can block interference with the electron transport chain in mitochondria induced by rotenone.

## 4. Discussion

The present study demonstrates that BV reduces rotenone-induced cell death in NSC34 cells by blocking the JNK and ERK1/2 signaling pathways. The MAPK family, including ERK1/2, stress-activated protein kinase/c-jun NH4-terminal kinase (SAPK/JNK), and p38 MAPKs [22,23], play central roles in the signaling pathways involved in cell proliferation, survival, and apoptosis. ERK1/2 are activated by mitogens and growth factors leading to cell growth and survival, whereas JNK and p38 MAPK are preferentially activated by pro-inflammatory cytokines and oxidative stress resulting in cell differentiation and apoptosis [24]. Here, we show that rotenone treatment causes decreased phosphorylation of ERK1/2 and increased phosphorylation of JNK. However, BV pretreatment induces the reduction of JNK and an increase of ERK1/2 activation in rotenone-treated NSC34 cells. After rotenone treatment, cell death was induced by the up-regulation of capase-3 activation and mitochondria aggregation leading to mitochondria dysfunction. However, BV treatment significantly suppressed the rotenone-induced activation of caspase-3 and mitochondria impairment in NSC34 cells. Taken those data together, we suggest that BV treatment could be useful for the inhibition of oxidative stress induced cell death in neurodegenerative diseases.

BV extracted from honeybees has been used in traditional oriental medicine. BV is traditionally used for the treatment of chronic inflammatory diseases such as rheumatoid arthritis and for relief of pain in oriental medicine [25]. In this study, BV attenuated rotenone-induced cell toxicity through inhibition of the JNK and activation of the ERK in neuronal cells. The optimized concentration of 2.5 μg/mL BV increased the expression of phospho-AKT [26]. The activation of MAPKs, including ERK, p38, and JNK [27,28], activated by interactions with a small GTPase and/or phosphorylation by protein kinases downstream from cell surface receptors. Previous reports have demonstrated that ERK is involved in growth-associated responses and cell differentiation. However, JNK and p38 are phosphorylated by environmental stresses, including the cytokine-like lipopolysaccharide (LPS) and oxidative stress [28].

Rotenone is used worldwide as an inhibitor of mitochondrial respiratory chain complex I, and induces PD-like symptoms in neurons through disrupting ATP supply [29]. Since it also increases ROS release and cause cell apoptosis, exposure of animals of rotenone induces dopaminergic cell death production [30,31].

Wagdy *et al.* have shown that BV acupuncture (BVA) has a neuroprotective effect against rotenone-induced oxidative stress, neuroinflammation, and cell death in PD-like animal model [32]. BV treatment inhibited rotenone-induced activation of caspase-3 and mitochondria aggregation inducing mitochondrial dysfunction. Those results provide that it is possible that multiple signaling pathways are involved in protective effects of BV, which deserves to be further investigated. Mitochondrial structure alternation by rotenone plays principal roles in cell death [33,34], which are critical pathological factors in neurodegenerative diseases including Alzheimer’s disease (AD), and amyotrophic lateral sclerosis (ALS) [35,36]. In further study, it is necessary to evaluate the effect of BV on genetically-engineered neurodegenerative disease animal models.
